# Treatment of Subcorneal Pustular Dermatosis (Sneddon-Wilkinson Disease) With Anti-Tumor Necrosis Factor Alpha

**DOI:** 10.7759/cureus.17147

**Published:** 2021-08-13

**Authors:** Clemence Guerin, Marie Beylot-Barry, Eric Frouin, Ewa Hainaut, Marie Masson Regnault

**Affiliations:** 1 Dermatology, Centre Hospitalier Universitaire de Poitiers, Poitiers, FRA; 2 Dermatology, Centre Hospitalier Universitaire de Bordeaux, Bordeaux, FRA; 3 Pathology, Centre Hospitalier Universitaire de Poitiers, Poitiers, FRA

**Keywords:** neutrophilic dermatosis, subcorneal pustular dermatosis, tnfα blocker, maintenance, relapse

## Abstract

Subcorneal pustular dermatosis (SPD), also known as Sneddon-Wilkinson disease, is a skin condition for which treatments are poorly codified. Anti-tumor necrosis factor alpha (TNFα) efficacy has been reported in multidrug-resistant SPD, as in our two cases.

In the first case, an 83-year-old woman was monitored for SPD, associated with monoclonal IgA gammopathy. After multiple-line treatment failure, infliximab (5mg/kg) led to clinical improvement, noted few days following the first injection, and with complete remission at one month. At 12 months, the patient relapsed and concomitant serum anti-TNFα antibodies were found. A switch to adalimumab led to complete remission in three months with a follow-up of six months.

In the second case, a 62-year-old woman was monitored for SPD associated with monoclonal IgA gammopathy recalcitrant to different lines of treatment. Treatment with adalimumab (40mg every two weeks) in combination with dapsone led to significant improvement after two injections. Five months later, she relapsed. It was then decided to reduce the interval between injections to once a week. Rapid improvement was achieved in one month allowing resumption of the original frequency of the injection without relapse after 20 months of follow-up.

In conclusion, our cases confirm the previously reported efficacy of anti-TNFα in resistant SPD. They also highlight a risk of secondary loss of efficacy, reinforced by the literature data. Substitution of another TNFα blocker or shortening of interval between injections provided a renewal in response to treatment.

## Introduction

Subcorneal pustular dermatosis (SPD) is a rare chronic neutrophilic dermatosis, described by Ian Sneddon and Darell Wilkinson in 1956 [1]. It more frequently affects women (sex ratio 4/1) [1,2] between 40 and 60 years of age and is characterized by large flaccid sterile pustules with hypopyon-like formations on the trunk, predominantly in intertriginous and flexion areas [3]. Histologic findings show a subcorneal pustule, almost resting on top of the epidermis, filled with polymorphonuclear neutrophils, with polymorphonuclear migration within the epidermis but without formation of spongiform pustules. Differential diagnosis, both clinical and histological, with generalized pustular psoriasis can be difficult. IgA monoclonal gammopathies are often found with SPD but other diseases such as rheumatoid arthritis, inflammatory bowel diseases, or more rarely some cancers or infections have also been reported [1,3-6]. First-line therapy is dapsone [1]. Other treatments can also be suggested such as corticosteroids [1], tetracyclines [1], colchicine [7], retinoids [1], or immunosuppressive drugs (methotrexate [5], cyclosporine [7], mycophenolate mofétil [8], azathioprine [8]). In multidrug-resistant SPD cases, anti-tumor necrosis factor alpha (TNFα) therapy has been suggested, with reported and documented efficacy in nine patients [3,5,7-12]. The long-term efficacy of these treatments has not been clearly established.

We describe two SPD patients initially successfully treated using anti-TNFα (adalimumab and infliximab), who quickly developed resistance to treatment, but finally recovered either by changing the molecule or reducing the interval between injections. 

## Case presentation

Case 1

**Figure 1:**
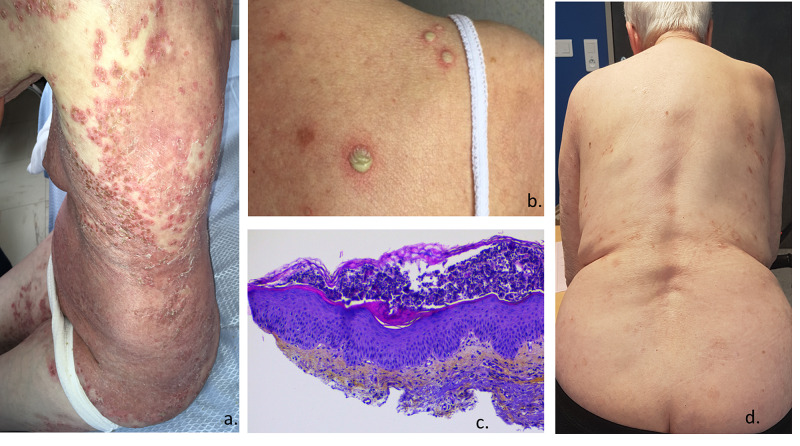
a. Well-delimitated erythemato-squamous plaques associated with pustules on the trunk. b. Hypopyon pustules. c. Skin biopsy: subcorneal pustulosis with neutrophilic infiltrated, H&E stain coloring (x10). d. Improvement of the lesions one month after infliximab.

An 83-year-old woman was referred to the department of dermatology for a rash that had appeared a month before. Her past medical history included ischemic heart disease and IgA monoclonal gammopathy. Physical examination showed erythematous and pruritic lesions coalescing to form well-delimited plaques on the trunk, intertriginous, and flexion areas (Figure 1a). The patient also had flaccid peripheral hypopyon-like pustules (Figure 1b) but reported no other symptoms. Complete blood count and hepatic and renal functions were all within normal limits. Plasma protein electrophoresis confirmed IgA monoclonal gammopathy, stable across time. Autoimmune results were negative. Biopsy revealed subcorneal pustulosis with suprabasal acantholysis and a mass of polymorphonuclear neutrophils without any spongiform pustule in the underlying epidermis (Figure 1c). Direct immunofluorescence was negative. SPD diagnosis in the context of IgA monoclonal gammopathy was established. After the failure of multiple lines of treatment including dapsone, cyclins, retinoids, methotrexate, azathioprine, salazopyrin, ultraviolet type B (UVB), and oral corticosteroids, infliximab (5mg/kg) was started, followed by additional injections at weeks 2 and 6 and subsequently every eight weeks. We observed spectacular clinical improvement within 48 hours, followed by a complete disappearance of the lesions after one month (Figure 1d). At twelve months, after the seventh treatment injection, some lesions reappeared at the end of the interval period. Reducing the interval between injections to six weeks was ineffective. The level of circulating infliximab was low (0.02mg/L) and the assay for quantification of infliximab antibodies was positive. We concluded that immunization against the treatment had occurred, explaining the secondary loss of response. A switch to adalimumab (40mg every two weeks) was decided, leading to complete remission at three months. Adalimumab was still effective six months after its introduction.

Case 2

**Figure 2:**
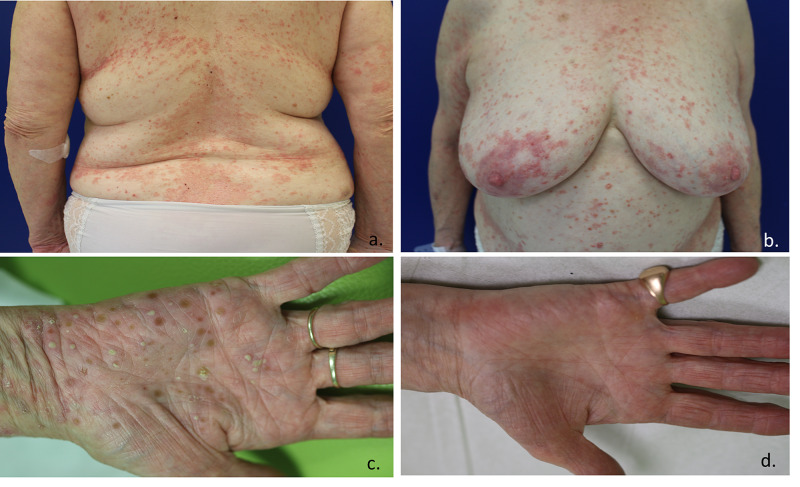
a, b. Erythematous plaques, with tendency to confluence with some crusting or scaling on the periphery on the trunk. c. Hypopyon pustules on the hand. d. Improvement of the lesions one month after adalimumab on the hand.

A 69-year-old woman whose past medical history included thyroid disorders presented with a rash that had been evolving for five years. An initial generalized pustular psoriasis diagnosis had led to treatment by methotrexate, followed by retinoids without any efficacy. Lesions appeared as well-defined erythematous, squamous, and pruritic plaques located on the trunk (Figure 2a, 2b), the loins, and the legs. Pustular lesions were noted in the hands (Figure 2c), the axillary area, and on the back forming a hypopyon, which resulted in an appearance resembling “half-half” blisters. The patient did not have any other related symptoms. Complete blood count and hepatic and renal functions were all within normal limits. Auto-antibody directed against the skin was negative. Plasma protein electrophoresis revealed IgA monoclonal gammopathy (2.34g/L). Biopsy revealed superficial pustular dermatosis with occasional eosinophils, without any supra-basal acantholysis. Direct immunofluorescence was negative. We retained the diagnosis of SPD associated with IgA monoclonal gammopathy. First-line therapy with dapsone followed by colchicine did not lead to any improvement. Corticosteroids were only temporarily effective. Adalimumab (40mg every two weeks) was started, associated with dapsone (50mg a day). We observed complete remission of the lesions after one month, and corticosteroids were consequently stopped (Figure 2d). Five months after treatment initiation, due to a relapse, we decided to reduce the interval between adalimumab injections to 40mg per week for one month. Clinical remission was attained, allowing resumption of the original frequency of the injection without relapse. After twelve months, clinical response was maintained without any possibility of interrupting dapsone treatment, as two previous attempts at this had resulted in gradual relapse.

## Discussion

While these observations confirm the previously reported efficacy of anti-TNFα in the SPD treatment, they also highlight the risk of secondary loss of efficacy.

SPD, also known as Sneddon-Wilkinson disease, is a rare, benign, yet relapsing pustular dermatosis. The differential diagnosis of SPD includes mainly pustular psoriasis, acute generalized exanthematous pustulosis (AGEP), and subcorneal type of IgA pemphigus. While AGEP and IgA pemphigus can easily be excluded, differential diagnosis of SPD or pustular psoriasis can be a challenge. Indeed, some authors even maintain that pustular psoriasis is closely related to SPD. Histological examination of a skin biopsy can be helpful in the diagnosis of SPD. The histologic feature is generally the subcorneal accumulation of neutrophils with the absence of spongiosis or acantholysis, although older lesions may show the latter as in one of our cases. In our two cases, we retained the diagnosis of SPD because of the large pustular lesions, the absence of typical histopathologic features of pustular psoriasis on the skin biopsies, and due to the association with monoclonal IgA gammopathy.

The physiopathology of SPD is not well-known, although TNFα seems to play a key role in SPD by recruiting, activating, and migrating polymorphonuclear neutrophils to form subcorneal pustules [1]. However, few studies have been published on the role of TNFα. Only one article has reported on one patient with pustules and high levels of TNFα in serum [13]. In the same article, ex vivo stimulation of the patient’s monocytes led to more production of anti-TNFα than in control patients’ monocytes [13]. Anti-TNFα efficacy and the association of SPD with others diseases involving TNFα (rheumatoid arthritis, inflammatory bowel disease) reinforce the hypothesis of TNF involvement in SPD. The literature review identified nine patients treated with anti-TNFα for SPD (Table 1) [3,5,7-12]. There were four women and five men, their mean age was 53.4 years (28 -78 years). the mean time of SPD evolution was 5.7 years (three weeks - 10 years). IgA gammopathy was diagnosed in two patients [3,12]. Treatment by anti-TNFα was introduced after the failure of an average of 6.2 lines of treatment (1-9 lines of treatment). Six patients were treated using infliximab, five by etanercept, and two by adalimumab. Initially, favorable evolution was described in all nine cases. The mean time for initial improvement was 41 days (1-120 days). The average follow-up after the introduction of treatment was 10.4 months (2-29 months). Relapse occurred in four patients with a median relapse time of eight months ( three to 29 months). Existing data suggest the usefulness of anti-TNFα in SPD treatment, although there probably exists a publication bias, as only successful results have been published up until now. 

**Table 1:** Literature review (and Case 1 and 2) of SPD treated by anti-TNFα. SLE: Systemic lupus erythematosus; OC: Oral corticosteroids; AH: Anti-histamine type 1; AZA: Azathioprine; LVEF: Left ventricular ejection fraction; MMF: Mycophenolate mofetil; MTX: Methotrexate; PUVA: Psoralen and ultraviolet A radiation; UVB: Ultraviolet type B phototherapy; TNFα: Tumor necrosis factor alpha.

Reference	Sex	Age (years)	Underlying disease	Duration	Previous treatment	Anti-TNFα	Time to 1^st^ improvement	Relapse	Time to relapse	Follow up
Case 1	F	83	IgA monoclonal gammopathy	11 years	dapsone, tetracycline, retinoids, MTX, AZA, sulfasalazine, UVB, OC	1. infliximab 5mg/kg	2 days	Yes	12 months	-
						2. adalimumab 40mg/2w	1 month	No	-	6 months
Case 2	F	69	IgA monoclonal gammopathy	7 years	MTX, acitretin, dapsone, colchicine, OC	1. adalimumab 40mg/2w	1 month	Yes	5 months	
						2. adalimumab 40mg/w 1 month then every 2w	1 month	No	-	12 months
Versini et al., 2013	M	78	IgA monoclonal gammopathy	7 years	dapsone, colchicine, acitretin, OC, PUVA, thalidomide, MTX	1. infliximab 5mg/kg	3 days	Yes	29 months	-
						2. etanercept 25mg x 2/w	3 weeks	No but alteration LVEF, discontinuation treatment	-	4 months
						3. adalimumab 40mg/2w	1 month	No	-	16 months
Naretto et al., 2009	F	37	SLE	5 years and 4 months	dapsone, OC, acitretin hydroxychloroquine, MMF, AZA, cyclosporin, colchicine	infliximab 5mg/kg	1 day	No	-	6 months
Bonifati et al., 2005	F	54	-	8 years	OC, acitretin, cyclosporin, colchicine, UVB, dapsone	infliximab 5mg/kg	2 days	Yes	3,5 months	-
Berk et al., 2008	M	51	-	6 years	acitretin, pimecrolimus, tazarotene, UVB, cefdinir, tetracycline, dapsone, colchicine, MTX	1. etanercept 50mg x 2/w	3 months	Yes	8 months	-
						2. etanercept 50mg x 2/w + acitrétine 25mg x 2/w	1 month	No	-	14 months
Berk et al., 2008	M	61	-	3 years	PUVA, ketoconazole, dapsone, colchicine, MMF, AH, OC	etanercept 50mg x 2/w	4 months	No	-	9 months
Bedi. 2007	F	28	-	10 years	OC, dapsone, tetracycline, nicotinamide, topical tacrolimus, colchicine	etanercept 25mg x2/w then etanercept 50mg x 2/w	1 month	No	-	11 months
Voigtlander et al., 2001	F	79	-	7 years	colchicine, acitretin, OC, UVB, AZA, azulfidine, dapsone	infliximab 5mg/kg + OC + acitretin	2 days	No (OC stopped)	-	6 months
Kretschmer et al., 2017	M	29	-	3 weeks	OC	infliximab 350mg single dose + dapsone	1 day	No (dapsone stopped, hemolytic anemia)	-	2 months
Blanchouin. 2009	M	64	IgA monoclonal gammopathy	5 years	dapsone, OC, PUVA, colchicine, thalidomide	1. infliximab	-	Yes	24 months	-
						2. etanercept	-	Yes	3 months	-
						3. adalimumab	-	No	-	18 months

Both our patients displayed IgA monoclonal gammopathy, associated with SPD, similarly to two cases reported in the literature [3,12]. The use of anti-TNFα in patients who received monoclonal gammopathy of undetermined significance (MGUS) diagnosis was not accompanied by any specific warnings [14]. A few rare cases of MGUS development during treatment by anti-TNFα have been documented [14]. Therefore, experts from Club Rhumatismes et Inflammation (CRI) suggest the use of anti-TNFα for patients carrying a known and stable gammopathy, under the condition that their plasma protein electrophoresis results are monitored regularly, initially every three months and subsequently every six months. Other authors have suggested a similar approach to monitoring, without specifying the frequency [14]. In both our cases, anti-TNFα did not worsen monoclonal gammopathy. 

Both of our patients developed resistance to treatment. In the first case, the presence of circulated infliximab antibodies indicated an auto-immunization against infliximab, which was what caused the loss of efficacy. This measurement was not performed in the second case or in any of the cases of the literature that showed clinical loss of efficacy. Production of anti-drug antibodies is not uncommon during anti-TNFα treatment [15]. In the context of psoriasis, for example, antibody prevalence was estimated variously depending on the studies, ranging from 5.4% to 43.6% for patients treated with infliximab, from 6% to 45% for patients treated with adalimumab, and from 0% to 18.3% for those treated with etanercept [15]. As regards neutralizing antibodies, their presence is associated with low or undetectable levels of circulating anti-TNFα and loss of clinical response [16,17]. Strategy for regaining response includes intensifying the treatment (either by increasing the doses or by reducing the interval between injections), changing to another anti-TNFα, or adding an immunomodulator/immunosuppressant [18]. The choice is guided by serum anti-TNFα and the presence and quantity of anti-TNFα antibodies [19]. In published cases of SPD showing a relapse during the use of anti-TNFα, management consisted in associating treatment with retinoids or corticosteroids, increasing anti-TNFα dosage, or changing anti-TNFα molecules, as we did in our first case, which led to the recovery of clinical response.

Finally, in an SPD case treated successfully using anti-TNFα, the levels of TNFα in the liquid in the pustules dropped, as did the levels of interleukins IL-6 and IL-8 [9]. Tocilizumab could also be an option in SPD treatment, as has been reported in one case [20].

## Conclusions

Anti-TNFα proves efficient in cases of multidrug-resistant SPD. In cases of secondary loss of response, management can be challenging but is guided by the measurement of the circulating level of anti-TNFα and the search for anti-drug antibodies. In one of our cases displaying loss of response due to circulating antibodies, change of anti-TNFα molecule led to the complete recovery of clinical response. In case of failure, cytokine inhibitors, including IL-6, could be considered as an option.
